# Supplementary material for: Dorsal raphe nucleus to anterior cingulate cortex 5-HTergic neural circuit modulates consolation and sociability
Source: eLife. 2021 Jun 3;10:e67638. doi: 10.7554/eLife.67638 (PMC8213405; doi:10.7554/eLife.67638)
Supplement: Transparent reporting form [file elife-67638-transrepform.pdf]

1st Floor
24 Hills Road
Cambridge CB2 1JP, UK

P 01223 855340
W elifesciences.org
T @elife

***eLife's* transparent reporting form**

We encourage authors to provide detailed information *within their submission* to facilitate the interpretation and replication of experiments. Authors can upload supporting documentation to indicate the use of appropriate reporting guidelines for health-related research (see EQUATOR Network), life science research (see the BioSharing Information Resource), or the ARRIVE guidelines for reporting work involving animal research. Where applicable, authors should refer to any relevant reporting standards documents in this form.

If you have any questions, please consult our Journal Policies and/or contact us: editorial@elifesciences.org.

Sample-size estimation

- You should state whether an appropriate sample size was computed when the study was being designed
- You should state the statistical method of sample size computation and any required assumptions
- If no explicit power analysis was used, you should describe how you decided what sample (replicate) size (number) to use

Please outline where this information can be found within the submission (e.g., sections or sfigure legends), or explain why this information doesn't apply to your submission:

This information can be found in the figure legends.

Figure 1 & 2. Optogenetic modulation of 5-HT neuron in the DR and 5-HT terminals in the ACC. *N* = 7–8 in each group. In the consolation test, independent samples t tests along with Bayesian independent samples t-test; in the open-filed and three-chamber test, two-way repeated measures ANOVA along with two-way repeated Bayesian measures ANOVA (light as within subject factor). The significant level was set at $P < 0.05$.

Figure 3. Chemogenetic modulation of DR 5-HT neuron activities in the DR→ACC neural circuit. *N* = 7–8 in each group, *P < 0.05, **P < 0.01 compared with vehicle control. For F, one tailed paired t test along with one tailed Bayesian paired samples t-test; for G-I, two-way repeated measures ANOVA along with two-way repeated Bayesian measures ANOVA.

Figure 4&5. Fiber photometry recording DR 5-HT neural dynamics and 5-HT release within the ACC during the consolation test. *N* = 5 in each group; for ' Δ F/F' value comparisons around the behaviors, paired samples t-test along with Bayesian paired samples t-test.

Figure 6. Intra-ACC injection of a 5-HT1AR agonist rescued sociability deficits induced by chemogenetic inhibition of DR 5-HT neurons in the DR→ACC neural circuit. *N* = 5–6 in each group, two-way ANOVA along with two-way Bayesian ANOVA along with Tukey *post -hoc* test.

Replicates

- You should report how often each experiment was performed
- You should include a definition of biological versus technical replication
- The data obtained should be provided and sufficient information should be provided to indicate the number of independent biological and/or technical replicates
- If you encountered any outliers, you should describe how these were handled
- Criteria for exclusion/inclusion of data should be clearly stated
- High-throughput sequence data should be uploaded before submission, with a private link for reviewers provided (these are available from both GEO and ArrayExpress)

Please outline where this information can be found within the submission (e.g., sections or figure legends), or explain why this information doesn't apply to your submission:

This information can be found in the Method section.

For Figure 1-3 and Figure 6, behavioral responses of each animal were scored and quantified just once. For Figure 4 and 5, fiber photometry signals were recorded only once unless a specific behavior occurred no more than four times during the 1-h test. In this case, the record was replicated in the following day.

For all the behavioral performance, some subjects show little or no movements, i.e., they just sited there during the test. We think these individuals did not show normal activity and motivation, therefore not appropriate to include their data in the following analysis. The sample sizes are '7' in some cases. (Source data file; Figure 1I: Male_ChR2 group, Female_ChR2 group; Figure 1L: Male_Dio group; Figure 2H: Male_Dio group; Figure 3G: Male_GI group; Figure 3H: Female_Dio group, Male_Gq_group, Male_GI group; Figure 3I: Female_Dio group, Male_Gq group, Male_GI_group).

Statistical reporting

- Statistical analysis methods should be described and justified
- Raw data should be presented in figures whenever informative to do so (typically when N per group is less than 10)
- For each experiment, you should identify the statistical tests used, exact values of N, definitions of center, methods of multiple test correction, and dispersion and precision measures (e.g., mean, median, SD, SEM, confidence intervals; and, for the major substantive results, a measure of effect size (e.g., Pearson's r, Cohen's d)
- Report exact p-values wherever possible alongside the summary statistics and 95% confidence intervals. These should be reported for all key questions and not only when the p-value is less than 0.05.

Please outline where this information can be found within the submission (e.g., sections or figure legends), or explain why this information doesn't apply to your submission:

This information can be found in the figure legends.

Statistical analysis methods, exact values of ‘N’, the distribution of the raw data had been provided in the figures and figure legends.

Raw data had been presented in the ‘source data’ document.

The detailed quantifications had been provided in Supplementary 2.

(For large datasets, or papers with a very large number of statistical tests, you may upload a single table file with tests, Ns, etc., with reference to sections in the manuscript.)

Group allocation

- Indicate how samples were allocated into experimental groups (in the case of clinical studies, please specify allocation to treatment method); if randomization was used, please also state if restricted randomization was applied
- Indicate if masking was used during group allocation, data collection and/or data analysis

Please outline where this information can be found within the submission (e.g., sections or figure legends), or explain why this information doesn’t apply to your submission:

This information can be found in the Method section.

During all the tests, all groups of experimental voles were randomly selected and the experimenters were blinded to the treatments.

Additional data files (“source data”)

- We encourage you to upload relevant additional data files, such as numerical data that are represented as a graph in a figure, or as a summary table
- Where provided, these should be in the most useful format, and they can be uploaded as “Source data” files linked to a main figure or table
- Include model definition files including the full list of parameters used
- Include code used for data analysis (e.g., R, MatLab)
- Avoid stating that data files are “available upon request”

Please indicate the figures or tables for which source data files have been provided:

As request, “Source data” files for all the figures had been provide along with the manuscript.
